# Reading Yule in light of the history and present of macroevolution

**DOI:** 10.1098/rstb.2023.0299

**Published:** 2025-02-20

**Authors:** Matt Pennell, Ailene MacPherson

**Affiliations:** ^1^ Department of Quantitative and Computational Biology, University of Southern California 90007, USA; ^2^ Department of Biological Sciences, University of Southern California 90007, USA; ^3^ Department of Computational Biology, Cornell University, Ithaca, NY 14850, USA; ^4^ Department of Mathematics, Simon Fraser University V5A 1S6, Canada

**Keywords:** macroevolution, phylogeny, palaeobiology, diversification

## Abstract

Yule’s 1925 paper introducing the branching model that bears his name was a landmark contribution to the biodiversity sciences. In his paper, Yule developed stochastic models to explain the observed distribution of species across genera and to test hypotheses about the relationship between clade age, diversity and geographic range. Here, we discuss the intellectual context in which Yule produced this work, highlight Yule’s key mathematical and conceptual contributions using both his and more modern derivations and critically examine some of the assumptions of his work through a modern lens. We then document the strange trajectory of his work through the history of macroevolutionary thought and discuss how the fundamental challenges he grappled with—such as defining higher taxa, linking microevolutionary population dynamics to macroevolutionary rates, and accounting for inconsistent taxonomic practices—remain with us a century later.

This article is part of the theme issue ‘"A mathematical theory of evolution": phylogenetic models dating back 100 years’.

## 1. Introduction

In 1925, George Udny Yule published a groundbreaking paper titled 'A mathematical theory of evolution, based on the conclusions of Dr. J. C. Willis, F.R.S’ [1] in the *Philosophical Transactions of the Royal Society*. This work introduced the model that later became known as the 'Yule process’, a stochastic model describing the growth and diversification of genera and species. The impact of this paper extends far beyond the fields we would now recognize as macroevolution, biogeography and macroecology, influencing the broader use of mathematical models in the life sciences and social sciences. In this perspective, we provide the intellectual context in which Yule developed his approach, explain his model in terms of more contemporary mathematical techniques and notation and discuss the influence his work had (and did not have) on the development of macroevolutionary thought, in particular. (We will touch briefly on other research areas where Yule’s paper influenced work, but only where they intersect with macroevolution.) We also highlight how some of the ideas Yule was wrestling with—the hierarchical nature of biodiversity, the role of taxonomy in shaping patterns of biodiversity and the effect of demography on speciation—are directly related to outstanding issues today.

In the early twentieth century, evolutionary theory was undergoing a period of intense debate and reevaluation. One of the central controversies during this time was the dispute between proponents of macromutational theories and those who advocated for gradualism. Macromutational theorists, such as de Vries [2,3], argued that new species could arise suddenly through mutations of large effect. Their intellectual opponents argued that the continuous variation observed in natural populations was more consistent with a gradual process of evolutionary change [4]. It was against this backdrop that J.C. Willis proposed his 'Age and Area’ hypothesis in his influential book of the same name [5]. In the book, Willis worked to understand the implications of macromutationism, which he supported for the distribution of biodiversity across space (de Vries also contributed to the volume). Willis posited that the geographic range of a species is determined by its age, with older genera having wider distributions and younger ones having more restricted ranges. This idea was supported both by first principles reasoning from a macromutationist perspective and by his own observations of plant species distributions, particularly on islands, as well as those of his contemporaries (e.g. [6,7]).

## 2. Yule’s models and empirical tests

Yule was not satisfied by Willis’ reasoning. Rather, he proposed that the relationship between the age of a species and its geographic distribution was likely a more complex result of the species’ age and its demography. At the time that Willis’ book was published, Yule was already an accomplished and influential applied mathematician. (For discussions of his contributions, see [8,9]; we note that Yule’s work in macroevolution warrants only one line of discussion in [9].) Yule, who had previously co-authored a paper with Willis on the distribution of biodiversity [10], felt that the Age and Area hypothesis did not make sense in light of Darwinian evolution:

*On the Darwinian view that species are continually dying out—that a species rises, flourishes and dies, superseded by the more advantageous form—a species occupying a very small area may be young, but it is equally likely or more likely to be old (a dying species). On Darwin’s own view that the whole body of individuals in a species becomes altered together, the young, species must be found occupying a large area at once, and the species occupying a small area could only be a ‘dying’ species. On the Darwinian view therefore either there need be no relation between Age and Area, or there would be a negative relation, species occupying small areas being on the whole the oldest. Similarly, on the Darwinian view a genus of a few, or of only one, species may be either young or old—a dying genus—and there need be no necessary relation between Age and Size. That species occupying very small areas, and the species of monotypic genera are mainly ‘relic’ forms, is, I gather, the predominant Darwinian view. Dr. Willis’s conclusions are inconsistent with that view.* [1, p.22]

More importantly, Yule recognized that a more rigorous, quantitative approach was required to generate meaningful hypotheses about the relationship between clade size, age and geographic distribution.

While there are many derivations of the 'Yule model’ available (e.g. [11,12]), these are often challenging to square with the actual models presented in his landmark paper. This is because how he formulated the problem, his notation and his mathematical techniques for solving the problem are all quite distinct from more modern approaches. Yule was interested in the relationship between the age of a clade (which he referred to as a genus) and the number of species in that clade. He viewed this as a demographic question involving the dynamics of individuals within a species, species within genera and birth of new genera through time. It was through this demographic lens that he referred to the 'age’ of a genus and the 'birth’ and 'death’ of species [1, p. 22]. Intriguingly, Yule’s demographic approach does not naturally require any explicit tree structure [13] (see e.g. [14]).

To bridge the gap between this original conceptualization versus modern uses and to better illustrate how Yule originally worked out his solution, we re-derive his results through a modern lens. We take two complementary approaches. First, we try to faithfully reproduce his modelling results using notation and terminology that will be more familiar to contemporary researchers. Second, we show how his derivations are different from later iterations on the same basic template that relied on innovations in stochastic process theory (following Bailey’s 1964 formulation [15]) and coalescent thinking (following Nee and colleagues' influential papers [16,17]); we put all three into a common notation so that the essential differences will be more apparent.

We start, as Yule did, with the simplest of scenarios. Yule considered a clade originating from a single 'primordial’ [1, p. 21] lineage at time t=0, which diversifies forward in time with species arising at a per capita rate λ between the origin and the present day, resulting in a clade of age (or, alternatively, ‘tree height’) T. Throughout we will use t to denote forward-time (from the origin t=0 to the present day t=T) as used by Yule and τ to denote backward-time (from the present τ=0 to the origin τ=T), as used in coalescent-style approaches; that is, whichever way you count time, the total time covered by the tree is the same. Yule assumed that all lineages are exchangeable [18] (also referred to as a 'single-type’ model [19]), such that the processes of diversification are identical for all lineages and that the population sizes of the constituent species do not influence the processes of diversification at all (we will return to both of these assumptions below). Given these assumptions, Yule [1, p. 36] considered the expected clade size $\bar{n}(T)$ and the probability that a clade has n lineages $P_{n}(T)$ at the present day (t=T). While the 'Yule model’ is understood today as a diversification process without extinction, Yule actually had a particular extinction scenario in mind, which he referred to as 'cataclysm’ [1, p. 80]. In other words, bad things happen to good lineages—land masses sink into the ocean, volcanoes erupt and rivers dry up. These are geologically instantaneous, they occur at random intervals and are, on average, non-selective. Of course, if a river dries up, all of the species that make their home there will be at risk of extinction, but the next time it happens, it will likely be a different river. Yule claimed that if these random events were the predominant mode of extinction, he could safely ignore them in the calculation of clade size. As later work has shown [15,17,20,21], this is not actually true and the quantities Yule derived are correct if the only process that changes clade size is speciation.

### (a) Yule’s probabilistic approach

To derive the mean and full distribution of clade sizes arising from a given primordial lineage, Yule [1, p. 33] divided the total time T into m small intervals of length $\Delta t=\frac{T}{m}$ such that the probability that a lineage speciates in a given unit of time is $p=\lambda \Delta t=\frac{\lambda T}{m}$ . The corresponding probability that a lineage does not speciate in an interval is then q=1−p and we assume that the probability that two speciation events occur in the same interval can be ignored (as it is of order $O(\Delta t^{2})$). The probability that there are n lineages in the clade at the present day then is given by the probability that *exactly* $n^{\prime }=n-1$ speciation events occurred. Each time a speciation event occurs, the number of lineages—and hence the total probability that a speciation event happens in the clade—increases. As such, we must consider the probability that the $n^{\prime }$ speciation events occur in the time intervals $j_{1},j_{2},\ldots ,j_{n^{\prime }}$, as well as the probability that speciation events do not occur between each of these times. For example, for the second speciation event to occur in interval $j_{2}$, neither of the lineages present can speciate between interval $j_{1}+1$ through interval $j_{2}-1$—an outcome that occurs with probability $q^{2(j_{2}-j_{1}-1)}$. In intervals where speciation events occur (for example, $j_{k}$) any one of the k species present can speciate and all of the other k−1 species must not speciate. Therefore, the probability of observing a single speciation event in an interval is $k(pq^{k-1})$. Carrying this forward, the resulting probability of observing n species after m intervals Pr⁡(n|m) is:


(2.1) $$\begin{array}{r} \begin{array}{r} \begin{array}{rl} & \mathrm{Pr}(n|m)=\\ & \sum_{j_{1}=1}^{m-n^{\prime }+1}\sum_{j_{2}=2}^{m-n^{\prime }}\ldots \sum_{j_{n^{\prime }}=n^{\prime }}^{m}\underset{1^{st}\;speciation\;event}{\underbrace{q^{j_{1}-1}p}}\;\underset{2^{nd}\;speciation\;event}{\underbrace{q^{2(j_{2}-j_{1}-1)}(2pq)}}\ldots \underset{n^{\prime }\;speciation\;event}{\underbrace{q^{n^{\prime }(j_{n^{\prime }}-j_{n^{\prime }-1}-1)}(n^{\prime }pq^{n^{\prime }-1})}}\underset{prob.\;of\;no\;more\;events}{\underbrace{q^{n(m-j_{n^{\prime }}-1)}}}, \end{array} \end{array} \end{array}$$

where the sums are over all the possible intervals in which each subsequent speciation event can occur.

Given that we have assumed that $\Delta t=\frac{T}{m}$ is small, we are interested in the limit as m→∞. To evaluate this limit, we note that $p=\frac{\lambda T}{m}$ and $q=1-\frac{\lambda T}{m}$. Using the fact that $\lim_{m\to \infty }{\left(1-\frac{\lambda T}{m}\right)}^{m}=e^{-\lambda T}$, we can obtain the expression for the probabilities of clades of size 1 through 4 given in table 1.

**Table 1.** Probabilities of observing n taxa after m intervals and after time T as the number of intervals goes to Infinity.

n	Pr⁡(n|m)	$P_{n}(T)=\lim_{m\to \infty }\;\mathrm{Pr}(n|m)$
1	$q^{m}$	$e^{-\lambda T}$
2	$\frac{pq^{m-1}\left(q^{m}-1\right)}{q-1}$	$e^{-\lambda T}\left(1-e^{-\lambda T}\right)$
3	$\frac{2p^{2}q^{m-2}\left(q^{m}-1\right)\left(q^{m}-q\right)}{\left(q-1{)}^{2}(q+1\right)}$	$e^{-\lambda T}{\left(1-e^{-\lambda T}\right)}^{2}$
4	$\frac{6p^{3}q^{m-3}\left(q^{m}-1\right)\left(q^{2m}-q^{m+1}-q^{m+2}+q^{3}\right)}{(q-1{)}^{3}(q+1)\left(q^{2}+q+1\right)}$	$e^{-\lambda T}{\left(1-e^{-\lambda T}\right)}^{3}$

Calculating these expressions and limits for larger n is arduous but they provided Yule [1, p. 36] and us with an intuition as to the general expression for the probability of observing n descendants of a single primordial lineage at present day time, T.


(2.2) $$\begin{array}{lcr} & P_{n}(T)=e^{-\lambda T}{\left(1-e^{-\lambda T}\right)}^{n-1}. & \end{array}$$

Using this probability, Yule [1, p. 36] also calculated the expected number of descendants at time T:


(2.3) $$\begin{array}{lcr} & \bar{n}(T)=E[n(T)]=\sum_{n=1}^{\infty }ne^{-\lambda T}{\left(1-e^{-\lambda T}\right)}^{n-1}=e^{\lambda T}. & \end{array}$$

This important result showed that the average clade size grows as we would expect under a deterministic model. Yule, surprisingly, did not consider the variance in n(T) among independent realizations of the evolutionary process, which is given by:


(2.4) $$\begin{array}{lcr} & \text{Var}(n(T))=E[(n(T){)}^{2}]-E[(n(T)){]}^{2}=e^{\lambda T}\left(e^{\lambda T}-1\right). & \end{array}$$

### (b) Deriving Yule’s result using stochastic processes

In 1964, Bailey [15] re-derived Yule’s core result above by conceptualizing diversification as a continuous-time stochastic process. As we discuss below, it was Bailey’s formulation of the problem that influenced David Raup’s pathbreaking work in palaeobiology [21]. We can express the Yule model in terms of a continuous-time stochastic process with states n={0,1,2,…} representing the number of species present in the clade at time t. The transition rate between state i∈ℕ and state j∈ℕ is then:


$$q_{i,j}=\left\{ \begin{array}{ll} -\lambda i & j=i\;\;\;\;\;no\;speciation\\ \;\;\;\lambda i & j=i+1\;speciation\\ \;\;\;0 & \text{otherwise} \end{array}\right.$$

Using these rates we can then examine clade size using a master equation approach—a system of ordinary differential equations describing the probability that a clade has n species at time t:


(2.5) $$\begin{array}{lcr} & \frac{dP_{n}(t)}{dt}=\sum_{j=0}^{\infty }q_{j,n}P_{j}(t)=-\lambda nP_{n}(t)+\lambda (n-1)P_{n-1}(t), & \end{array}$$

where $P_{0}(t)=0$ (the probability the clade goes extinct) given that we assume there is at least one 'primordial lineage’ and no extinction (i.e. we only need to consider n,i,&j>0 in the Yule model). Assuming, as Yule did, that there is a single primordial species the initial condition for this system gives:


$$P_{n}(0)=\left\{ \begin{array}{ll} 1 & n=1\\ 0 & \text{otherwise}. \end{array}\right.$$

To solve this system of ODEs in two different ways, we can either follow Yule and solve successively for the cases of small n, identify a pattern in the solutions and propose a general expression, or we can follow [15] and obtain the general solution from generating functions (not shown here). While we think that Bailey’s master equation approach is significantly easier to understand than Yule’s probabilistic approach, it is hard to generalize to more complex diversification models because the system can be difficult or impossible to solve in these cases. The lesson that evolutionary biology would learn from the development of Coalescent Theory in the 1980s [22] is that when faced with a hard problem, it is often easier to spin it around and solve it backwards.

### (c) Deriving Yule’s result using a coalescent-style approach

In our view, Nee and colleagues’ [16,17] formulation of the problem makes it even more straightforward to understand what Yule was doing than either Yule’s own or Bailey’s subsequent one. To see why, let τ be the time before the present day, such that τ=0 is the present and τ=T is the time at which the clade originates. Let $P_{n}(\tau )$ be the probability that a single species present at time τ in the past gives rise to n descendants at the present day. In the manner of Nee *et al*. [17], we can consider the possible events that can occur to a focal lineage going backward in time between τ and τ+Δτ. Like Yule, we assume the time interval Δτ is small enough that the probability that two events occur in an interval can be neglected. In this case, there are only two outcomes: either nothing happens (with probability 1−λΔτ), or a speciation event occurs (with probability λΔτ). Looking backward in time, a speciation event involves the joining of two lineages. If we wish the ancestor of this speciation event to have n offspring, then we must observe a speciation event that joins one lineage with j≤n descendants and another lineage with n−j descendants [23]. As such,


(2.6) $$\begin{array}{lcr} & P_{n}(\tau +\Delta \tau )=\underset{\text{nothing happens}}{\underbrace{\left(1-\lambda \Delta \tau \right)P_{n}(\tau )}}+\underset{\text{speciation event}}{\underbrace{\sum_{j=0}^{n}\lambda \Delta \tau P_{j}(\tau )P_{n-j}(\tau )}}+O(\Delta \tau^{2}). & \end{array}$$

Subtracting $P_{n}(\tau )$ from both sides, dividing by Δτ and taking the limit as Δτ→0, we can use the definition of the derivative to obtain the system of backward-in-time master equations:


(2.7) $$\frac{d}{d\tau }P_{n}(\tau )=-\lambda P_{n}(\tau )+\sum_{j=0}^{n}\lambda P_{j}(\tau )P_{n-j}(\tau ).$$

These master equations have the convenient initial condition:


$$P_{n}(0)=\left\{ \begin{array}{cc} 1 & n=1\\ 0 & \text{otherwise} \end{array}\right.$$

as each lineage observed at the present has, by definition, exactly one descendant. Noting that $P_{n}(T)=P_{n}(T)$ (where $P_{n}(T)$ is defined as in the previous section), this system of ODEs can be solved recursively to obtain the distribution in equation 2.2.

### (d) Yule’s empirical analyses and their interpretation

Yule used the mathematical model to do a number of things. Among them was simply to compare the distribution of genera sizes generated by his model with those of empirical compilations of genera size for four taxonomic groups: snakes, lizards and two groups of beetles. Yule was hesitant for the reader to over-interpret his results ('If any conclusions stated in this Introduction or in the body of the paper seem to be too confident, or at all dogmatic, I hope the reader will attribute the appearance to inadvertence of wording, or a simple desire to avoid the wearisome reiteration of qualifying phrases’ [1, p. 32]). But he also cannot help but be impressed with how well his model matches empirical data:

*So far as the tests go I think it must be admitted that the formula given is capable of representing the facts with considerable precision, more closely indeed than we have any right to expect. One might well have expected the personal factor in classification, the practically cataclysmic destruction of species at numerous epochs in geological time, and all the varied changes that have diversified the organic history of our planet, to have left so many irregularities in the distribution that any formula could at most have given a very rough analogy with the general run of the data. But apparently the formula arrived at can do far more than this.* [1, p. 58]

One interpretation of this result is that much of the variation in group sizes we observe can be attributed to stochasticity alone: one need not invoke the creator’s inordinate fondness for any particular group of organisms. In thinking in these terms, Yule was more than fifty years ahead of his time (see e.g. refs [24,25], discussed below) in appreciating the role of chance in generating major patterns of biodiversity. However, for reasons that we discuss in §5a, we should take this empirical result with a shaker of salt—and the same applies to his attempt to address Willis’ hypothesis regarding the correlation between age and geographic area that inspired this work in the first place.

## 3. Yule’s work had little impact on the early development of macroevolutionary biology

Reading Yule’s paper today is a jarring experience. While the presentation of the work is certainly consistent with that of his time, the style of analysis and broader way in which he thinks of the problem seem right at home in the macroevolutionary literature of the late twentieth century. And this is consistent with the remarkable fate of Yule’s paper; although it is widely recognized today as being a foundational contribution to the study of biodiversity, it had remarkably little influence on the development of our field for much of its history. For one, George Gaylord Simpson, the principal representative of palaeontology in the modern synthesis and the pioneer of the study of taxonomic rates, did not seem even aware of Yule’s work. Not only is Yule not cited in either of his two influential books on macroevolution [26,27], but Simpson does not place much emphasis on the importance of stochasticity or in developing null models for the sizes of the clades he is studying.

In the 1970s, the study of palaeontology underwent an incredible transformation—this new 'palaeobiology’ was quantitative and theory-driven. (We refer interested readers to David Sepkoski’s riveting historical work [28] on the topic.) One of the key intellectual projects of the 'palaeobiological revolution’ was that of the MBL group (named for their meetings at the Marine Biological Laboratory at Woods Hole). The MBL group—a collaboration between palaeobiologists David Raup, Stephen Jay Gould, Thomas Schopf and the ecologist Dan Simberloff—developed a series of simulation models, which were used to generate null distributions for the patterns of diversity under purely stochastic processes [29–32]. They saw this work as a foundation for a 'nomothetical’ (law-forming) palaeobiology; by comparing the patterns of idealized clades to those of the fossil record, they hoped to uncover a set of rules that could explain the ebb and flow of biodiversity [28]. Whether one judges their project a success or not, it undoubtedly did have lasting impact on palaeobiology, as well as on their intellectual descendants who primarily used molecular phylogenies, in orienting the fields around the idea that we should expect a wide range of outcomes from the processes of diversification and one needs to do statistical tests to compare observed data with these expectations. In retrospect, this work seems in line with how Yule was approaching similar problems in the 1920s, with the distinction that Yule was, like molecular phylogeneticists many years later, focused on a snapshot in time, rather than the complete history. Throughout the 1970s and early 1980s, the MBL researchers were unaware of Yule’s work in this area (and indeed, we suspect that the MBL researchers could have saved some precious computing resources if they had known about Yule’s analytical approaches for generating clade size distributions). As if to drive this omission home, Schopf wrote a lengthy paper [24] examining the history of thought on and study of stochasticity in generating macroevolutionary patterns. Schopf seemingly touches on every relevant work (as well as many only tangentially relevant ones)—except that of Yule. And in a similar vein, Leigh van Valen [33] famously used the exponential distribution of species durations to argue for the central importance of Red Queen dynamics between hosts and parasites. However, exponential branch lengths also are expected to arise from the models of chance developed by Yule, yet van Valen does not consider this (see also [34] on this point).

And this is not some quirk of Yule’s paper being missed by a few key palentologists. David Kendall, who made a foundational contribution to the theory of birth–death processes [20] and whose paper is now routinely cited in conjunction with Yule, seems unaware of his work (at least at the time he was first working on this problem; see [13]). (We believe that the original derivation of the birth–death process was by Feller [35], who also did not cite Yule.) Neither does Kendall mention Yule’s contribution in his historical survey 'Branching processes since 1863’ [36]. The mammalogist Sydney Anderson revisited the claims [37] of Age and Area and developed his own stochastic model of clade evolution; despite providing a lengthy discussion of many previous attempts to explain the distribution of clade sizes, he only mentions Yule’s paper off-handedly and does not engage with the actual model or results.

We do not have a good explanation for Yule’s pioneering work being overlooked for so long—it was occasionally cited by prominent biodiversity scientists, including Preston [38] and the above-mentioned Anderson [37] so it was not as if it was completely missed in these fields. And as noted in §1, Yule was well-known during his time and subsequently. We do think is it notable, however, that by the time macroevolution had come around to thinking about the problem of species diversity in a quantitative way that was reminiscent of Yule’s approach, the theory of stochastic processes and the use of Monte Carlo simulations were well developed. It is only obvious in retrospect that Yule’s model could be welded to these two to gain insight into the processes that have driven patterns of biodiversity.

## 4. The rediscovery of Yule

One macroevolutionary researcher who did eventually see the brilliance of Yule’s contribution was Raup [39]. As noted above, Raup was one of the key members of the MBL group, though he eventually diverged from the other members [28]. In his 1978 paper 'Cohort analysis of generic survivorship’ notes that [a]n important but generally overlooked paleontological application [of branching processes] is that of Yule [1]’ ([40, p. 5); to the best of our knowledge, this is the first time that Raup discussed Yule’s work. Two of his contributions in the prior year [32,41] on closely related topics did not mention Yule or Bailey’s [15] text, which we suspect was where he first encountered Yule’s work on the topic, given how extensively Raup leans on Bailey’s work. By the time he wrote his 1985 paper 'Mathematical models of cladogenesis’ [21]—one of the most influential papers in the history of macroevolution—he prominently placed Yule’s work as a foundational contribution in a long and illustrious intellectual lineage applying branching processes to biology. Following this work, researchers developed increasingly sophisticated rate estimators for fossil occurrence data [42–44] and, as described above, Nee and colleagues [17] demonstrated that diversification rates could be estimated from emerging dated molecular phylogenies. It is hard to overstate the importance of this contribution, not only in shaping modern diversification analyses (e.g. [45–47]) in macroevolution but also in epidemiology [19,48] and fields like cell biology [49].

It is important to emphasize however that this is only part of a broader and more complicated story. In the contemporary intellectual landscape, statistical phylogenetics and macroevolution are deeply intertwined fields [50,51] . But this was not the case for most of the latter part of the twentieth century, where there were multiple distinct research traditions along similar themes that were not in close conversation with one another. So while we focus on Yule’s belated influence on macroevolutionary thought (i.e. on the research tradition that developed from palaeobiology and extends to this day), his model also influenced the development of the first statistical models for reconstructing phylogenetic trees (see for instance, seminal contributions by Cavalli-Sfroza and Edwards [52], Edwards [53] and Thompson [54] in the 1960s and early 1970s). And some mathematicians studying stochastic processes had recognized Yule’s model even earlier [14,15]. As these formerly disparate fields began to coalesce in the 1980s through the early twenty-first century, there was a patchwork of historical traditions; in some of these Yule’s work was preeminent and in others, it was not. For example, in a 1989 paper [25], the phylogenetic biologists Slowinksi and Guyer weave together many different threads on the history of using taxonomic and phylogenetic data to test for the effect of stochasticity in macroevolution, yet they leave Yule out of the picture entirely. In his influential 2001 paper [55] on phylogenetic models for tree shape, Aldous discusses Yule at length but does not mention the contributions of Nee and colleagues [16,17] more than half a decade earlier, nor does a recent summary by Fischer and colleagues [56] of an extensive literature on tree balance statistics [e.g. 56–63].

## 5. Contemporary questions in macroevolution that Yule anticipated

To close this perspective, we wanted to highlight some additional issues and challenges that Yule raised in his 1925 paper that anticipated current debates in the field of macroevolution. As we show below, we do not think that his perspectives were always right but it is truly remarkable that he was engaged with these topics at a time when the field essentially did not yet exist.

### (a) The hierarchical nature of diversification

*The possible effect of size of genus (number of species in the genus) on the chance of a generic mutation is also ignored. This assumption may or may not be correct, but was deliberate. The generic characters are regarded as representing a main position of stability, and the chance of occurrence of a transfer from one main position of stability to another is regarded as independent of the number of minor positions of stability (species) which may have been taken up within the main position (genus)* [1, p. 24]*.*

We devoted our explanation of Yule’s model to describing how he computed the probability distribution for sizes of clades of age T. This model, which forms the basis of much future work, was nested in a larger, justly forgotten model, even though we think the core idea was interesting. Recall that Yule was operating in the intellectual context where macromutationism was widely seen as a viable hypothesis. He deduced that there were two types of mutations: 'generic mutations’—large effect mutations that produced the synapomorphies that defined clades—and 'specific mutations’, which give rise to new speciation events. As he noted in the quote above, he considered these to be independent nested processes, perhaps akin to the processes that generated Simpson’s adaptive zones [27] and the subsequent diversification within these. Today, most researchers studying lineage evolution consider all splits in the tree as representing speciation events, rather than as a combination of different types of events, including those that lead to higher taxonomic units. Palaeobiologists often use the more inclusive term 'origination rate’ to describe the process of lineage splitting because it is more agnostic regarding the taxonomic status of the newly formed groups [42], but nonetheless they only consider a single process. However, there have been some recent attempts to follow Yule’s approach; that is, to treat higher taxonomic groups as real evolutionary units and describe the processes by which they are formed [64–67].

But in the broader sense, Yule’s conceptualization of diversification is a particular example of a broad category of models in which diversification occurs at different paces among different types [19]; in Yule’s model, the two types are genera and species, which each have a distinct origination rate. The 'multi-type models’ most widely used today are 'state-dependent speciation and extinction’ (SSE) models pioneered by Maddison and colleagues [46], which capture variation in diversification rates among lineages [68–71]. We can use the terminology of modern multi-type models to understand how Yule conceptualized diversification, how it stands in contrast to SSE models and why many of his mathematical results are incorrect. We begin by noting three key differences between Yule’s formulation of the problem and modern SSE models. First, Yule’s state space (i.e. the number of types) was infinite—while each species was labelled as belonging to a genus, there was no limit to the number of genera that could be present. Second, while SSE models assign different evolutionary rates to each type in Yule’s model, all species regardless of genera diversified at the rate *λ*. Third, since new genera were formed by an irreversible transition (*sensu* [72]) occurring at rate *γ* in an existing lineage, this meant that most of the genera (except those that had no descendant genera) were paraphyletic. The widespread acceptance of the phylogenetic systematics approach to classification in the latter part of the twentieth century (see Hull [73]) likely precluded the exploration of similar types of models.

For the single-type case, Yule calculated the probability that a genus has n species given that it arose from a single 'primordial’ lineage T time units ago. The hierarchical multi-type model described above allowed Yule to consider a complementary measure of genus size, the probability that a *randomly sampled genus* at time T has n species, which we denote as Pr⁡(n|T). The sampled genus may be the primordial lineage itself or a newly formed genus arising from a generic mutation sometime between t=0 and t=T. Averaging the genus size over the range of possible genus ages, a, we have by the law of total probability:


(5.1) $$\begin{array}{lcr} & \mathrm{Pr}(n|T)=\int_{0}^{T}\mathrm{Pr}(a|T)\;\mathrm{Pr}(n|a,T)da, & \end{array}$$

where Pr⁡(n|a,T) is calculated as


(5.2) $$\begin{array}{lcr} & \mathrm{Pr}(n|a,T)=\mathrm{Pr}(n|a)=e^{-\lambda a}{\left(1-e^{-\lambda a}\right)}^{n-1}. & \end{array}$$

Note that this assumes that Pr⁡(n|a,T) is independent of the total age of the tree T. Equation 5.2 is analogous to the probability calculated in equation 2.2.

This leaves us to derive an expression for the probability that a randomly sampled genus at the present day T arose at time T−a and hence is of age a. Note that the age of a genus, a, cannot be older than the whole tree, T, hence the limits of the integral. If T is small then the sampled genus is likely to be the primordial genus, whereas if T is large, most genera will have arisen since the origin of the tree and Pr⁡(a|T) will depend solely on when generic mutations occurred. Recognizing this, Yule broke his analyses down into a transient case for small T and a limiting case for large T. We focus only on the limiting case here as the probability Pr⁡(a|T) is in fact far more difficult to calculate than Yule proposed.

To understand how Yule proposed to calculate this quantity, let m be the number of genera at the present day and $\tilde{m}$ the number of genera present at time T−a when the focal genus arose. Noting that the probability of a generic mutation occurring between time T−a and T−a+dt is $\gamma \tilde{m}\;dt$, Yule [1, p. 37] approximated this probability of a genus having age a as:


$$\mathrm{Pr}(a|T)=\frac{E[\gamma \tilde{m}]}{E[m]},$$

where the expectations are over all stochastic realizations of the diversification process. There are several logical problems with this expression. First m and $\tilde{m}$ and not independent; for example, trees with $\tilde{m}$ lineages at time T−a in the past must have at least $m>\tilde{m}$ lineages at the present day. More subtly, the number of lineages at the present day is also not independent of whether or not a generic mutation occurred at time T−a. A modern backward-in-time approach could be used to calculate Pr⁡(a|T) correctly, but doing so is neither useful, given the biological limitations of Yule’s model, nor straightforward. We hope the reader will attribute our omission to a 'simple desire to avoid the wearisome’ derivations.

### (b) Taxonomic patterns reflect both biological realities and scientific conventions

*The desire of the systematist to break up a genus which he regarded as unwieldy might well tend to cause a deficiency of very large genera, but such a deficiency can hardly be held to be proved by the present tables* [1, p. 58]

Here, Yule is prescient about the potential confounding effect of what he elsewhere in the essay (quoted above) refers to as the 'personal factor in classification’—the fact that species counts per genus or the number of genera in a higher group are a consequence of both biology and human decisions—even if he thought it was not a significant issue for his own analysis. Macroevolutionary biologists, both working with extinct [74,75] and extant taxa [76,77] have long shared Yule’s concern that inferences about diversification might be misled by biases in taxonomic practice. One might think that such issues become moot when looking at phylogenetic trees of lineages rather than Linnaean hierarchies—indeed, this is one of the motivations for tree-based, rather than rank-based taxonomic systems [78]—but this is not the case [79]. We necessarily look at subsets of the Tree of Life when conducting comparative analyses, and these subtrees often tend to be especially large or especially diverse ecologically, which might lead to biased inferences of typical rates [80,81]; looking only at small trees has its downsides too [82]. And in practice, researchers tend to use taxonomic labels [45] to subset the tree. The nodes that we label with higher taxonomic classifications are viewed as special by systematicists (i.e. species that descend from a named node may harbor evolutionarily significant synapomorphies, for instance); the branch length distributions corresponding to trees stemming from named nodes may be different from random nodes on the Tree of Life [83–85]. And furthermore, the problem of how to define terminal taxa never went away. This is particularly pernicious if the way we define lineages systematically biases the distribution of node ages. For example, bacterial ‘species’ are typically defined using thresholds of sequence divergence and this can lead to a concentration of splitting events at certain times (see [86,87], for attempts to grapple with this bias). Another challenging situation is when taxa are defined inconsistently across an ecological gradient that we think might drive diversification. For example, a number of recent studies have found that recent speciation rates are faster in the temperate regions than the tropics [88], which goes against the predictions of classic theories for the existence of the latitudinal diversity gradient [89]. However, these findings are potentially undermined if biological species are often more narrowly defined in the better studied temperate regions [90]. We anticipate that in the near future, researchers will be reckoning with how best to make inferences about the processes of diversification that are not so confounded with taxonomic practice.

### (c) The relationship between population dynamics and macroevolutionary rates

*At first, as the species spreads, the number of individuals must tend to increase. But over the very long periods which have to be considered there must be a countervailing tendency to ultimate decrease in the number of individuals, owing to the increase in the number of species. The area available being limited, the tendency, as it seems to me, must be towards greater and greater numbers of species and fewer individuals in each. Possibly the fact that ignoration of the number of individuals leads to results in close accordance with the data may only indicate this tendency first to increase and then to decrease in the size of the species* [1, p. 24]*.*

One notable aspect of reading Yule’s paper in light of contemporary research is how much space he devoted to justifying assuming a Poisson model to describe the speciation process; today, this assumption is overwhelmingly taken for granted and rarely justified, even though the macromutational model of speciation is no longer taken seriously as it was in Yule’s time. One way Yule justified this was pure ignorance; he figured that, in reality, speciation rates should be a function of population size, but did not know what type of function to specify. Yule’s intuition as to what this might look like is remarkably close to the canonical neutral theory of biodiversity and biogeography developed decades later [91,92]. Neutral theory assumes that the number of individuals in an ecosystem is fixed, such that as the number of species in a clade increases, the average number of individuals per species must decrease, a concept known as the 'zero-sum assumption’ [91,93]. However, if one works out the implications of using such an assumption on the resulting phylogenetic trees, they look very different from those generated by Yule’s original model as well as empirical ones [94]; some researchers argue that incorporating ‘protracted speciation’, where speciation happens as a prolonged process and not as an instantaneous point mutation, mitigates these failures [95]. We refer readers to Kopp [96] for a more in-depth discussion of the point mutation model of speciation in the context of neutral theory. We editorialize, though, that neutral theory has likely received more criticism about its speciation process than related approaches that tend not to make their assumptions explicit (e.g. standard birth–death models in macroevolution can be extended to include protracted speciation [97,98] but this is rarely considered in practice).

The neutral theory stands out as a rare attempt to actually model the relationship between speciation rate and population size. This is a particularly challenging problem since the rate limiting step for completing speciation is thought to be the persistence of nascent species [88,99–101], rather than the rate at which incipient species form; if this is the case, then it is not just the absolute population size that matters for speciation rates but also the population dynamics of these newly separated populations. Given this complexity, most of the current frontier of modeling approaches rely on simulations (e.g. refs. [102–106]), which can allow speciation to emerge from complex eco-evolutionary dynamics, but when one wants to actually fit the model to data to test hypotheses about the causes of evolutionary rate variation, we must still typically resort to the standard branching processes that Yule was working with (but for exceptions, see [107,108]). This is an obvious point for future development but given how little progress we have made in the past 100 years, one that will obviously be difficult.

## 6. Concluding remarks

Yule’s model is now firmly in the pantheon of core evolutionary models [109], sitting alongside the Wright–Fisher process [110,111], the Moran process [112] and Kingman’s coalescent [22]. As such, it hardly needs our promotion. Nonetheless, in this paper we have tried to make two cases. First, that it was far from inevitable that Yule would be viewed as a forebear of modern macroevolution; the field developed largely without his influence and his ingenuity was only belatedly recognized in this area (as we noted, it was recognized earlier in adjacent fields of study). Second, that Yule’s insights into the *process* of diversification were extraordinarily prescient given what was known about speciation biology in his time: the problems he was wrestling with as he was thinking through his model are still some of the most pressing challenges in modern macroevolution. We also tried to highlight some the ways in which his conceptualization of the problem is distinct from our current ways of thinking and some limitations in his calculations. We hope that our paper is both a helpful guide and a little nudge for researchers who, like ourselves until embarrassingly recently, have left Yule’s paper perpetually sitting on their 'to read’ pile.

## Data Availability

This article has no additional data.

## References

[1] Yule GU. 1924 II.—A mathematical theory of evolution, based on the conclusions of Dr. J. C. Willis, F. R. S. Phil. Trans. R. Soc. Lond. B **213**, 21–87. (10.1098/rstb.1925.0002)

[2] de Vries H. 1901 Die mutationstheorie. Leipzig, Switzerland: Veit & comp.

[3] Vries H de, MacDougal DT. 1906 Species and varieties; their origin by mutation. Chicago, IL: Open Court Pub. Co. (10.5962/bhl.title.32555)

[4] Provine WB. 1971 The Origins of Theoretical Population Genetics. Chicago, IL: University of Chicago Press.

[5] Willis JC. 1922 Age and area: a study in geographical distribution and origin of species. Cambridge, UK: The University Press. (10.5962/bhl.title.70451)

[6] Reid C. 1899 The origin of the British flora. London, UK: Dulau. (10.5962/bhl.title.24157)

[7] Guppy HB, Guppy HB. 1903 Observations of a naturalist in the Pacific between 1896 and 1899. New York, NY: MacMillan & Co. Ltd. (10.5962/bhl.title.56077)

[8] Tabery JG. 2004 The "Evolutionary Synthesis’’ of George Udny Yule. J. Hist. Biol. **37**, 73–101. (10.1023/b:hist.0000020390.75208.ac)

[9] Yates F. 1952 George Udny Yule 1871–1951. Obit. Not. Fellows R. Soc. **8**, 308–323. (10.1098/rsbm.1952.0020)

[10] Willis JC, Yule GU. 1922 Some Statistics of Evolution and Geographical Distribution in Plants and Animals, and their Significance. Nature **109**, 177–179. (10.1038/109177a0)

[11] Durrett R. 2012 Essentials of stochastic processes. New York, NY: Springer. (Springer texts in statistics). (10.1007/978-1-4614-3615-7)

[12] Steel M, McKenzie A. 2001 Properties of phylogenetic trees generated by Yule-type speciation models. Math. Biosci. **170**, 91–112. (10.1016/s0025-5564(00)00061-4) 11259805

[13] Lambert A. 2024 Ages, sizes and (trees within) trees of taxa and of urns, from Yule to today. Phil. Trans. R. Soc. B **380**, 20230305. (10.1098/rstb.2023.0305)39976410

[14] Simon HA. 1955 On a Class of Skew Distribution Functions. Biometrika **42**, 425. (10.2307/2333389)

[15] Bailey NTJ. 1964 The elements of stochastic processes with applications to the natural sciences. London, UK; New York, NY: John Wiley & Sons.

[16] Nee S, Mooers AO, Harvey PH. 1992 Tempo and mode of evolution revealed from molecular phylogenies. Proc. Natl. Acad. Sci. **89**, 8322–8326. (10.1073/pnas.89.17.8322) 1518865 PMC49910

[17] Nee S, May RM, Harvey PH. 1994 The reconstructed evolutionary process. Phil. Trans. R. Soc. Lond. B **344**, 305–311. (10.1098/rstb.1994.0068) 7938201

[18] Stadler T, Bonhoeffer S. 2013 Uncovering epidemiological dynamics in heterogeneous host populations using phylogenetic methods. Phil. Trans. R. Soc. B **368**, 20120198. (10.1098/rstb.2012.0198) 23382421 PMC3678323

[19] MacPherson A, Louca S, McLaughlin A, Joy JB, Pennell MW. 2021 Unifying Phylogenetic Birth-Death Models in Epidemiology and Macroevolution. Syst. Biol. **71**, 172–189. (10.1093/sysbio/syab049) 34165577 PMC8972974

[20] Kendall DG. 1948 On the Generalized “Birth-and-Death” Process. Ann. Math. Stat. **19**, 1–15. (10.1214/aoms/1177730285)

[21] Raup DM. 1985 Mathematical models of cladogenesis. Paleobiology **11**, 42–52. (10.1017/s0094837300011386)

[22] Kingman JFC. 1982 On the genealogy of large populations. J. Appl. Probab. **19**, 27–43. (10.2307/3213548)

[23] Louca S, McLaughlin A, MacPherson A, Joy JB, Pennell MW. 2021 Fundamental Identifiability Limits in Molecular Epidemiology. Mol. Biol. Evol. **38**, 4010–4024. (10.1093/molbev/msab149) 34009339 PMC8382926

[24] Schopf TJM. 1979 Evolving paleontological views on deterministic and stochastic approaches. Paleobiology **5**, 337–352. (10.1017/s0094837300006606)

[25] Slowinski JB, Guyer C. 1989 Testing the Stochasticity of Patterns of Organismal Diversity: An Improved Null Model. Am. Nat. **134**, 907–921. (10.1086/285021)

[26] Simpson GG. 1984 Tempo and mode in evolution. New York, NY: Columbia University Press. (10.7312/simp93040)

[27] Simpson GG. 1953 The major features of evolution. New York, NY: Columbia University Press. (10.7312/simp93764)

[28] Sepkoski D. 2012 Rereading the fossil record: the growth of paleobiology as an evolutionary discipline. Chicago, IL: University of Chicago Press.

[29] Raup DM, Gould SJ, Schopf TJM, Simberloff DS. 1973 Stochastic Models of Phylogeny and the Evolution of Diversity. J. Geol. **81**, 525–542. (10.1086/627905)

[30] Raup DM, Gould SJ. 1974 Stochastic Simulation and Evolution of Morphology-Towards a Nomothetic Paleontology. Syst. Zool. **23**, 305. (10.2307/2412538)

[31] Schopf TJM, Raup DM, Gould SJ, Simberloff DS. 1975 Genomic versus morphologic rates of evolution: influence of morphologic complexity. Paleobiology **1**, 63–70. (10.1017/s0094837300002207)

[32] Gould SJ, Raup DM, Sepkoski JJ Jr, Schopf TJM, Simberloff DS. 1977 The shape of evolution: a comparison of real and random clades. Paleobiology **3**, 23–40. (10.1017/s009483730000508x)

[33] van Valen L. 1973 A New Evolutionary Law. Evol. Theory **1**, 1–30.

[34] Raup DM. 1975 Taxonomic survivorship curves and Van Valen’s Law. Paleobiology **1**, 82–96. (10.1017/s0094837300002220)

[35] Feller W. 1939 Die Grundlagen der Volterraschen Theorie des Kampfes ums Dasein in wahrscheinlichkeitstheoretischer Behandlung. Acta Biotheor. **5**, 11–40. (10.1007/bf01602932)

[36] Kendall DG. 1966 Branching Processes Since 1873. J. Lond. Math. Soc. **s1-41**, 385–406. (10.1112/jlms/s1-41.1.385)

[37] Anderson S. 1974 Patterns of Faunal Evolution. Q. Rev. Biol. **49**, 311–332. (10.1086/408171) 4477905

[38] Preston FW. 1948 The Commonness, And Rarity, of Species. Ecology **29**, 254–283. (10.2307/1930989)

[39] German RZ. 2009 21.Intellectual Evolution Across an Academic Landscape. In The paleobiological revolution (eds D Sepkoski, M Ruse), pp. 416–422. Chicago, IL: University of Chicago Press. (10.7208/chicago/9780226748597.003.0022)

[40] Raup DM. 1978 Cohort analysis of generic survivorship. Paleobiology **4**, 1–15. (10.1017/s0094837300005649)

[41] Raup DM. 1977 Stochastic Models in Evolutionary Palaeontology. In Developments in palaeontology and stratigraphy patterns of evolution as illustrated by the fossil record (ed. A Hallam), pp. 59–78. Leipzig, Switzerland: Elsevier. (10.1016/s0920-5446(08)70323-6)

[42] Foote M. 2000 Origination and extinction components of taxonomic diversity: General problems. Paleobiology **26**, 74–102. (10.1017/s0094837300026890)

[43] Foote M. 2003 Origination and Extinction through the Phanerozoic: A New Approach. J. Geol. **111**, 125–148. (10.1086/345841)

[44] Silvestro D, Schnitzler J, Liow LH, Antonelli A, Salamin N. 2014 Bayesian Estimation of Speciation and Extinction from Incomplete Fossil Occurrence Data. Syst. Biol. **63**, 349–367. (10.1093/sysbio/syu006) 24510972 PMC4361715

[45] Magallón S, Sanderson MJ. 2001 Absolute diversification rates in angiosperm clades. Evolution **55**, 1762–1780. (10.1111/j.0014-3820.2001.tb00826.x) 11681732

[46] Maddison WP, Midford PE, Otto SP. 2007 Estimating a binary character’s effect on speciation and extinction. Syst. Biol **56**, 701–710. (10.1080/10635150701607033) 17849325

[47] Alfaro ME, Santini F, Brock C, Alamillo H, Dornburg A, Rabosky DL, Carnevale G, Harmon LJ. 2009 Nine exceptional radiations plus high turnover explain species diversity in jawed vertebrates. Proc. Natl. Acad. Sci. USA **106**, 13410–13414. (10.1073/pnas.0811087106) 19633192 PMC2715324

[48] Stadler T *et al*. 2012 Estimating the Basic Reproductive Number from Viral Sequence Data. Mol. Biol. Evol. **29**, 347–357. (10.1093/molbev/msr217) 21890480

[49] Stadler T, Pybus OG, Stumpf MPH. 2021 Phylodynamics for cell biologists. Science **371**, h6266. (10.1126/science.aah6266) 33446527

[50] O’Meara BC. 2012 Evolutionary Inferences from Phylogenies: A Review of Methods. Annu. Rev. Ecol. Evol. Syst. **43**, 267–285. (10.1146/annurev-ecolsys-110411-160331)

[51] Pennell MW, Harmon LJ. 2013 An integrative view of phylogenetic comparative methods: connections to population genetics, community ecology, and paleobiology. Ann. N. Y. Acad. Sci. **1289**, 90–105. (10.1111/nyas.12157) 23773094

[52] Cavalli-Sforza LL, Edwards AWF. 1967 Phylogenetic analysis: Models and estimation procedures. Evolution **21**, 550–570. (10.1111/j.1558-5646.1967.tb03411.x) 28563688

[53] Edwards AW. 1970 Estimation of the Branch Points of a Branching Diffusion Process. J. R. Stat. Soc. **32**, 155–164.

[54] Thompson EA. 1975 Human evolutionary trees. Cambridge, UK: Cambridge University Press.

[55] Aldous DJ. 2001 Stochastic models and descriptive statistics for phylogenetic trees, from Yule to today. Stat. Sci. 23–34 **16**. (10.1214/ss/998929474)

[56] Fischer M, Herbst L, Kersting SJ, Kühn AL, Wicke K. 2023 Extension of tree balance and imbalance indices and alternative ways of measuring tree balance. In Tree balance indices: a comprehensive survey, pp. 341–344. Cham, Switzerland: Springer International Publishing. (10.1007/978-3-031-39800-1_25)

[57] Kirxpatrick M, Slatkin M. 1993 Searching for evolutionary patterns in the shape of a phylogenetic tree. Evolution **47**, 7. (10.1111/j.1558-5646.1993.tb02144.x) 28564277

[58] Mooers AO, Heard SB. 1997 Inferring Evolutionary Process from Phylogenetic Tree Shape. Q. Rev. Biol. **72**, 31–54. (10.1086/419657)

[59] Blum MGB, François O. 2006 Which Random Processes Describe the Tree of Life? A Large-Scale Study of Phylogenetic Tree Imbalance. Syst. Biol. **55**, 685–691. (10.1080/10635150600889625) 16969944

[60] Aldous DJ, Krikun MA, Popovic L. 2011 Five Statistical Questions about the Tree of Life. Syst. Biol. **60**, 318–328. (10.1093/sysbio/syr008) 21386112

[61] Jones GR. 2011 Tree Models for Macroevolution and Phylogenetic Analysis. Syst. Biol. **60**, 735–746. (10.1093/sysbio/syr086) 21865338

[62] Colijn C, Gardy J. 2014 Phylogenetic tree shapes resolve disease transmission patterns. Evol. Med. Public Health **2014**, 96–108. (10.1093/emph/eou018) 24916411 PMC4097963

[63] Kersting SJ, Wicke K, Fischer M. 2024 Tree balance in phylogenetic models. Phil. Trans. R. Soc. B **380**, 20230303. (10.1098/rstb.20230303)39976413

[64] Foote M. 2012 Evolutionary dynamics of taxonomic structure. Biol. Lett **8**, 1070. (10.1098/rsbl.2012.0780) PMC3259952 21865239

[65] Maruvka YE, Shnerb NM, Kessler DA, Ricklefs RE. 2013 Model for macroevolutionary dynamics. Proc. Natl. Acad. Sci. USA **110**, E2460–E2469. (10.1073/pnas.1220014110) 23781101 PMC3703965

[66] Humphreys AM, Barraclough TG. 2014 The evolutionary reality of higher taxa in mammals. Proc. R. Soc. B **281**, 20132750. (10.1098/rspb.2013.2750) PMC3996593 24695424

[67] Humphreys AM, Rydin C, Jønsson KA, Alsop D, Callender‐Crowe LM, Barraclough TG. 2016 Detecting evolutionarily significant units above the species level using the generalised mixed Yule coalescent method. Methods Ecol. Evol. **7**, 1366–1375. (10.1111/2041-210x.12603)

[68] Morlon H, Parsons TL, Plotkin JB. 2011 Reconciling molecular phylogenies with the fossil record. Proc. Natl. Acad. Sci. USA **108**, 16327–16332. (10.1073/pnas.1102543108) 21930899 PMC3182748

[69] Goldberg EE, Igić B. 2012 Tempo and mode in plant breeding system evolution. Evolution **66**, 3701–3709. (10.1111/j.1558-5646.2012.01730.x) 23206129

[70] Beaulieu JM, O’Meara BC. 2016 Detecting Hidden Diversification Shifts in Models of Trait-Dependent Speciation and Extinction. Syst. Biol. **65**, 583–601. (10.1093/sysbio/syw022) 27016728

[71] Rasmussen DA, Stadler T. 2019 Coupling adaptive molecular evolution to phylodynamics using fitness-dependent birth-death models. eLife **8**, 45562. (10.7554/elife.45562) PMC6715349 31411558

[72] Goldberg EE, Igić B. 2008 On phylogenetic tests of irreversible evolution. Evolution **62**, 2727–2741. (10.1111/j.1558-5646.2008.00505.x) 18764918

[73] Hull DL. 2019 Science as a process: an evolutionary account of the social and conceptual development of science. Chicago, IL: University of Chicago Press.

[74] Alroy J. 2002 How many named species are valid? Proc. Natl. Acad. Sci. USA **99**, 3706–3711. (10.1073/pnas.062691099) 11891342 PMC122588

[75] Hendricks JR, Saupe EE, Myers CE, Hermsen EJ, Allmon WD. 2014 The Generification of the Fossil Record. Paleobiology **40**, 511–528. (10.1666/13076)

[76] Raikow RJ. 1986 Why are There so Many Kinds of Passerine Birds? Syst. Zool. **35**, 255. (10.2307/2413436)

[77] Minelli A, Fusco G, Sartori S. 1991 Self-similarity in biological classifications. Biosystems **26**, 89–97. (10.1016/0303-2647(91)90040-r) 1841641

[78] de Queiroz K, Gauthier J. 1994 Toward a phylogenetic system of biological nomenclature. Trends Ecol. Evol. **9**, 27–31. (10.1016/0169-5347(94)90231-3) 21236760

[79] Faurby S, Eiserhardt WL, Svenning J. 2016 Strong effects of variation in taxonomic opinion on diversification analyses. Methods Ecol. Evol. **7**, 4–13. (10.1111/2041-210x.12449)

[80] Pennell MW, Sarver BAJ, Harmon LJ. 2012 Trees of Unusual Size: Biased Inference of Early Bursts from Large Molecular Phylogenies. PLoS One **7**, e43348. (10.1371/journal.pone.0043348) 22957027 PMC3434155

[81] Louca S, Henao‐Diaz LF, Pennell M. 2022 The scaling of diversification rates with age is likely explained by sampling bias. Evolution **76**, 6. (10.1111/evo.14515) 35567800

[82] Beaulieu JM, O’Meara BC. 2018 Can we build it? Yes we can, but should we use it? Assessing the quality and value of a very large phylogeny of campanulid angiosperms. Am. J. Bot. **105**, 417–432. (10.1002/ajb2.1020) 29746717

[83] Henao-Diaz LF, Pennell M. 2023 The Major Features of Macroevolution. Syst. Biol. **72**, 1188–1198. (10.1093/sysbio/syad032) 37248967

[84] Rabosky DL. 2009 Ecological Limits on Clade Diversification in Higher Taxa. Am. Nat. **173**, 662–674. (10.1086/597378) 19302027

[85] Stadler T, Rabosky DL, Ricklefs RE, Bokma F. 2014 On Age and Species Richness of Higher Taxa. Am. Nat. **184**, 447–455. (10.1086/677676) 25226180

[86] Morlon H, Kemps BD, Plotkin JB, Brisson D. 2012 Explosive radiation of a bacterial species group. Evolution **66**, 2577–2586. (10.1111/j.1558-5646.2012.01598.x) 22834754 PMC3871994

[87] Louca S, Shih PM, Pennell MW, Fischer WW, Parfrey LW, Doebeli M. 2018 Bacterial diversification through geological time. Nat. Ecol. Evol. **2**, 1458–1467. (10.1038/s41559-018-0625-0) 30061564

[88] Schluter D, Pennell MW. 2017 Speciation gradients and the distribution of biodiversity. Nature **546**, 48–55. (10.1038/nature22897) 28569797

[89] Mittelbach GG *et al*. 2007 Evolution and the latitudinal diversity gradient: speciation, extinction and biogeography. Ecol. Lett. **10**, 315–331. (10.1111/j.1461-0248.2007.01020.x) 17355570

[90] Freeman BG, Pennell MW. 2021 The latitudinal taxonomy gradient. Trends Ecol. Evol. **36**, 778–786. (10.1016/j.tree.2021.05.003) 34074540

[91] Hubbell SP. 2001 The unified neutral theory of biodiversity and biogeography. Princeton, NJ: Princeton University Press.

[92] Bell G. 2001 Neutral Macroecology. Science **293**, 2413–2418. (10.1126/science.293.5539.2413) 11577228

[93] Rosindell J, Hubbell SP, Etienne RS. 2011 The Unified Neutral Theory of Biodiversity and Biogeography at Age Ten. Trends Ecol. Evol. **26**, 340–348. (10.1016/j.tree.2011.03.024) 21561679

[94] Nee S. 2005 The neutral theory of biodiversity: do the numbers add up? Funct. Ecol. **19**, 173–176. (10.1111/j.0269-8463.2005.00922.x)

[95] Rosindell J, Cornell SJ, Hubbell SP, Etienne RS. 2010 Protracted speciation revitalizes the neutral theory of biodiversity. Ecol. Lett. **13**, 716–727. (10.1111/j.1461-0248.2010.01463.x) 20584169

[96] Kopp M. 2010 Speciation and the neutral theory of biodiversity: Modes of speciation affect patterns of biodiversity in neutral communities. Bioessays **32**, 564–570. (10.1002/bies.201000023) 20586053

[97] Etienne RS, Rosindell J. 2012 Prolonging the Past Counteracts the Pull of the Present: Protracted Speciation Can Explain Observed Slowdowns in Diversification. Syst. Biol. **61**, 204. (10.1093/sysbio/syr091) 21873376 PMC3280041

[98] Etienne RS, Morlon H, Lambert A. 2014 Estimating the duration of speciation from phylogenies. Evolution **68**, 2430–2440. (10.1111/evo.12433) 24758256 PMC4262007

[99] Rosenblum EB, Sarver BAJ, Brown JW, Des Roches S, Hardwick KM, Hether TD, Eastman JM, Pennell MW, Harmon LJ. 2012 Goldilocks Meets Santa Rosalia: An Ephemeral Speciation Model Explains Patterns of Diversification Across Time Scales. Evol. Biol. **39**, 255–261. (10.1007/s11692-012-9171-x) 22707806 PMC3364415

[100] Dynesius M, Jansson R. 2014 Persistence of within-species lineages: A neglected control of speciation rates. Evolution **68**, 923–934. (10.1111/evo.12316) 24329123

[101] Harvey MG, Singhal S, Rabosky DL. 2019 Beyond Reproductive Isolation: Demographic Controls on the Speciation Process. Annu. Rev. Ecol. Evol. Syst. **50**, 75–95. (10.1146/annurev-ecolsys-110218-024701)

[102] Gascuel F, Ferriere R, Aguilee R, Lambert A. 2015 How Ecology and Landscape Dynamics Shape Phylogenetic Trees. Syst. Biol. **64**, 590–607. (10.1093/sysbio/syv014) 25771083

[103] Rosindell J, Harmon LJ, Etienne RS. 2015 Unifying ecology and macroevolution with individual‐based theory. Ecol. Lett. **18**, 472–482. (10.1111/ele.12430) 25818618 PMC4403962

[104] Manceau M, Lambert A, Morlon H. 2015 Phylogenies support out‐of‐equilibrium models of biodiversity. Ecol. Lett. **18**, 347–356. (10.1111/ele.12415) 25711418

[105] Aguilée R, Gascuel F, Lambert A, Ferriere R. 2018 Clade diversification dynamics and the biotic and abiotic controls of speciation and extinction rates. Nat. Commun. **9**, 3013. (10.1038/s41467-018-05419-7) 30068945 PMC6070539

[106] Rangel TF, Edwards NR, Holden PB, Diniz-Filho JAF, Gosling WD, Coelho MTP, Cassemiro FAS, Rahbek C, Colwell RK. 2018 Modeling the ecology and evolution of biodiversity: Biogeographical cradles, museums, and graves. Science **361**, r5452. (10.1126/science.aar5452) 30026200

[107] Pontarp M, Brännström Å, Petchey OL. 2019 Inferring community assembly processes from macroscopic patterns using dynamic eco‐evolutionary models and Approximate Bayesian Computation (ABC). Methods Ecol. Evol. **10**, 450–460. (10.1111/2041-210x.13129)

[108] Overcast I *et al*. 2021 A unified model of species abundance, genetic diversity, and functional diversity reveals the mechanisms structuring ecological communities. Mol. Ecol. Resour. **21**, 2782–2800. (10.1111/1755-0998.13514) 34569715 PMC9297962

[109] Cohen JE. 2004 Mathematics Is Biology’s Next Microscope, Only Better; Biology Is Mathematics’ Next Physics, Only Better. PLoS Biol. **2**, e439. (10.1371/journal.pbio.0020439) 15597117 PMC535574

[110] Fisher RA. 1923 XXI.---On the Dominance Ratio. Proc. R. Soc. Edinb. **42**, 321–341. (10.1017/S0370164600023993)

[111] Wright S. 1931 Evolution in Mendelian populations. Genetics **16**, 97–159. (10.1093/genetics/16.2.97) 17246615 PMC1201091

[112] Moran PAP. 1958 Random processes in genetics. Math. Proc. Camb. Philos. Soc. **54**, 60–71. (10.1017/s0305004100033193)

